# Geographic Variations in the Incidence of Glioblastoma and Prognostic Factors Predictive of Overall Survival in US Adults from 2004–2013

**DOI:** 10.3389/fnagi.2017.00352

**Published:** 2017-11-07

**Authors:** Hao Xu, Junrui Chen, Hongzhi Xu, Zhiyong Qin

**Affiliations:** Department of Neurosurgery, Huashan Hospital Shanghai Medical College, Fudan University, Shanghai, China

**Keywords:** ethnicity, geographic region, glioblastoma, prognosis, Surveillance, Epidemiology, End Results (SEER) Program

## Abstract

**Objective:** The purpose of this study was to evaluate variations in the regional incidence of glioblastoma in US adults in 2004–2013.

**Study Design and Setting:** We evaluated 24,262 patients with primary glioblastoma. Data were categorized based on geographic regions that included different SEER registry sites as follows: (1) Northeast: Connecticut, New Jersey (3,977 patients); (2) South: Kentucky, Louisiana, Metropolitan Atlanta, Rural Georgia, Greater Georgia (excluding AT and RG) (5,212 patients); (3) North Central: Metropolitan Detroit, Iowa (2,320 patients); (4) West: Hawaii, New Mexico, Seattle (Puget Sound), Utah, San Francisco-Oakland SMSA, San Jose-Monterey, Los Angeles, Greater California (excluding SF, LA, and SJ), Alaska (12,753 patients).

**Results:** Statistically significant differences in the rates of overall patient survival (*P* < 0.001) and the incidence of glioblastoma (24.31, 22.6, 20.35, 15.03 per 100,000/year in the South, Northeast, West, North Central regions, respectively) were identified between geographic regions. Multivariate Cox regression analysis demonstrated that overall survival was better in patients of Asian or Pacific Islander race. In addition, age, registry site, marital status, tumor laterality, histological classification, the extent of disease, tumor size, tumor extension, and treatment methods were identified as significant prognostic factors.

**Conclusion:** Glioblastoma incidence is geographic region and race/ethnicity–dependent.

## Introduction

In the United States, primary malignant brain tumors are rare and account for about 2% of all adult cancers (American Cancer Society, 2012). Despite their rarity, brain cancer incidence has increased over the last 30 years while survival rates remain extremely poor (Deorah et al., 2006). Glioblastoma is one of the most common and highly invasive malignant brain neoplasms with an incidence of 2–3 new cases per 100,000 people per year worldwide (ICBTRotUS, 2012). Due to its aggressiveness, the median survival time for a newly diagnosed patient is approximately 1 year, with < 5% of patients surviving 5 years post-diagnosis (Aldape et al., 2003; Reuss and von Deimling, 2009; ICBTRotUS, 2012).

Once diagnosed, patients typically undergo surgical resection followed by adjuvant radiotherapy and chemotherapy (Ryu et al., 2014; Huang et al., 2017). The tumor's highly aggressive behavior, resistance to the adjuvant therapy as well as the diffuse and invasive nature of the neoplasm (Sang, 2016) result in very few long-term survivors (Adamson et al., 2009). Research aiming to understand the molecular mechanisms of glioblastoma progression revealed its highly heterogeneous nature of genetic alterations creating an obstacle for the development of targeted treatments (Dunn et al., 2012). Effective novel approaches such as immunotherapy (Thomas et al., 2012), gene therapy (Brown et al., 2016), and oncolytic virus therapy (Markert et al., 2000; Jiang et al., 2009; Wollman et al., 2012) along with the strategies using bacteria-mediated drug delivery (Mehta et al., 2016), autophagy inhibition (Levy et al., 2017), tumor-treating fields technology (Stupp et al., 2015) and using polymeric nanofibres to guide tumor cells to cytotoxic hydrogel (Jain et al., 2014) are being evaluated. Although very promising, the efficacy of many of these therapies or their combinations relies on a better understanding of the molecular mechanisms that drive cancer progression (Reuss and von Deimling, 2009). However, only understanding of specific causes underlying glioblastoma formation and growth may lead to the development of curative specific treatments to complement the current standard of care.

Despite the abundant research, our knowledge about specific causes for glioblastoma development is still very limited. Exposure to ionizing radiation, rare genetic mutations, and family history are the accepted risk factors for brain tumors; however, only a small proportion of brain malignancies is attributable to these risk factors (Fisher et al., 2007). Other potential risk factors like cell phone use, smoking, and environmental exposures have been studied, however, the conclusions were not definitive (Gomes et al., 2011). In addition, investigation of the patient's lifestyle, diet, occupation, blood group and history of head trauma did not result in meaningful associations (Zampieri et al., 1994). An inverse correlation seems to be present between the glioblastoma incidence and susceptibility to allergies, indicating an immunologic component in disease progression (Hochberg et al., 1990).

Glioblastoma incidence is gender and race-dependent. Glioblastoma is 1.6 times more common in men than women (Wen and Kesari, 2008; Ivan et al., 2012), and two to three times more common among the Caucasian than the black populations, American Indians, Alaskan Natives, and Asian-Pacific Islanders race groups (Ohgaki and Kleihues, 2005).

Cancer incidence varies among different geographic regions (Schwartzbaum et al., 2006). It was reported that there is an approximately 4-fold difference in the incidence of primary malignant brain tumors between countries with high incidence, such as Australia, Canada, Denmark, Finland, New Zealand and the US, and territories with low incidence, such as Rizal in the Philippines and Mumbai in India (Wrensch et al., 2002). Even in the USA, the glioblastoma incidence varies from state to state (Ostrom et al., 2016). In 2011, the age-adjusted brain and spinal tumor incidence for the United States were 6.4 per 100,000 people, and state incidences ranged from 3.4 to 10.3 (Howlader et al., 2016). Despite some evidence of regional differences in glioblastoma incidence, there is no clear understanding how geographic factors contribute to the development of this disease. A better understanding of the regional differences in glioma incidence and outcomes can increase awareness and may lead to improved protocols for glioblastoma detection and management in high-risk regions. In addition, identification of regional risk factors may suggest underlying mechanisms of tumor development and aid in prevention and treatment selection, ultimately improving the survival rates. Therefore, the aim of our study is to further explore and update regional glioblastoma incidence as well as factors influencing overall patient survival during years 2004–2013.

## Materials and methods

### Data source

The study involved a retrospective evaluation of medical records from Surveillance, Epidemiology, and End Results (SEER) Program (www.seer.cancer.gov) Research Data (2004–2013), (National Cancer Institute, 2016) DCCPS, Surveillance Research Program, Surveillance Systems Branch, released April 2016, based on the November 2015 submission. SEER is a population-based registry sponsored by the National Cancer Institute. It collects data on cancer incidence and survival from 18 geographic areas in the US, including approximately 30% of the US population (2016). SEER contains de-identified data, and analysis of the data does not require IRB approval or informed consent from patients. We have got permission to access the research data file in the SEER program by National Cancer Institute, USA with the reference number 12749-Nov2015^*****^-August 2016.

### Study population

Patients diagnosed with glioblastoma multiforme (GBM) of the brain and other regions of the nervous system (ICD-O-3 of C71.0-C71.4, C71.7-C71.9, and C72.0-C72.5) between 2004 and 2013 were included in the analysis. Histologic types were limited to glioblastoma, NOS (not otherwise specified) (ICD-O-3: 9440/3), giant cell glioblastoma (ICD-O-3: 9441/3), and gliosarcoma (ICD-O-3: 9442/3). Data were categorized based on geographic regions (northeast, south, north central, west). These regions include different SEER registry sites as follows: **(1) Northeast**: Connecticut, New Jersey; **(2) South**: Kentucky, Louisiana, Metropolitan Atlanta, Rural Georgia, Greater Georgia (excluding AT and RG); **(3) North Central**: Metropolitan Detroit, Iowa; **(4) West**: Hawaii, New Mexico, Seattle (Puget Sound), Utah, San Francisco-Oakland SMSA, San Jose-Monterey, Los Angeles, Greater California (excluding SF, LA and SJ), Alaska.

### Study variables

The first endpoint of the present study was glioblastoma incidence between 2004 and 2013 in 4 described regions of SEER registry sites. The second endpoint was overall survival (OS). It was calculated from the day of diagnosis to the date of death, which was indicated as “Vital Status” in the SEER database.

The variables obtained for each case included patient demographics (age at diagnosis, gender, marital status, insurance status, race/ethnicity), disease characteristics (laterality, histologic subtypes, extent of disease, tumor size, tumor extension, metastasis at diagnosis), and treatment modalities (no treatment, surgery, radiotherapy, and both surgery and radiation treatment).

### Statistical analysis

Comparability among four registry sites was tested using Chi-square test for categorical variables and analysis of variance (ANOVA) for continuous variables. Categorical data were represented by a number (n) and percentage (%) and continuous variables were represented as the mean and standard deviation (SD). The incidence rate of glioblastoma was calculated per 100,000 persons per year, and direct age adjustment was made to the population of the USA in 2000. The Kaplan-Meier method with log-rank test was used to compare overall survival (OS) among the registry sites. Univariate and multivariate Cox proportional hazard regression models were used for analysis of prognosis factors for survival outcomes. Variables that showed a tendency of association with OS (*P* < 0.05) in univariate analysis were evaluated using a multivariate Cox proportional hazard regression model with stepwise selection. All P values were two-sided and *P* < 0.05 were considered statistically significant. Statistical analyses were performed using the statistical software package SPSS version 22 (IBM, Armonk, NY).

## Results

### Characteristics of study subjects

**Figure 1:**
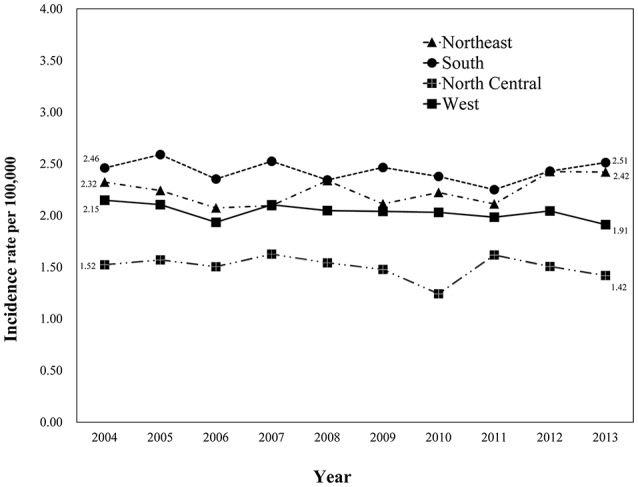
Variation in the incidence rates of glioblastoma among SEER registry sites between 2004 and 2013. Eligible patients diagnosed with primary glioblastoma registered by SEER database between 2004 and 2013 were categorized into four geographic regions [(1) Northeast, Connecticut, New Jersey; (2) South, Kentucky, Louisiana, Metropolitan Atlanta, Rural Georgia, Greater Georgia (excluding AT and RG); (3) North Central, Metropolitan Detroit, Iowa; (4) West, Hawaii, New Mexico, Seattle (Puget Sound), Utah, San Francisco-Oakland SMSA, San Jose-Monterey, Los Angeles, Greater California (excluding SF, LA and SJ), Alaska]. Age-adjusted glioblastoma incidence was calculated for each region with the highest incidence rate being among the patients from the South region (24.31 per 100,000/year), and the lowest being in the North Central region (15.03 per 100,000/year).

We identified 24,262 eligible patients with primary glioblastoma in SEER database registered between 2004 and 2013. There were 3,977 patients from the North (16.4%), 5,212 patients from the South (21.5%), 2,320 patients from the North Central (9.6%) and 12,753 patients from the West (52.6%) US regions. *Age*-*adjusted* glioblastoma *incidence*s were calculated for patients from different geographic regions (Figure 1). The highest incidence rate was among patients from the South region (24.31 per 100,000/year), followed by patients from the Northeast (22.36 per 100,000/year), the West (20.35 per 100,000/year) and the North Central region (15.03 per 100,000/year).

A comparison of the demographics and pathological features stratified by registry site is shown in Table 1. There were statistically significant differences in age, race, marital status, insurance status, tumor laterality, the extent of disease, tumor size, an extension of tumor, and treatments among patients from different regions (all *P* < 0.001). Patients from the North Central region had the highest mean diagnostic age (63.2 ± 14.7 years). Patients from the North Central region were more likely to be married (87.6%) and insured (90.2%), and had a lower rate of unilateral glioblastoma (77.9%).

**Table 1:** Basic demographics and clinical characteristics of patients with glioblastoma between 2004 and 2013 (*n* = 24,262), stratified by registry site.

	**Northeast (*n* = 3,977)**	**South (*n* = 5,212)**	**North Central (*n* = 2,320)**	**West (*n* = 12,753)**	***P*-value**
Diagnostic age (years)	63.1 ± 14.6	61.5 ± 14.6	63.2 ± 14.7	61.9 ± 14.8	<0.001^*^
**GENDER**, ***n*** **(%)**					
Male	2,242 (56.4)	2,994 (57.4)	1,308 (56.4)	7,396 (58.0)	0.211
Female	1,735 (43.6)	2,218 (42.6)	1,012 (43.6)	5,357 (42.0)	
**RACE**, ***n*** **(%)**					
White	3,665 (92.2)	4,524 (86.8)	2,102 (90.6)	11,351 (89.2)	<0.001^*^
Black	199 (5.0)	632 (12.1)	187 (7.9)	1,428 (5.9)	
American Indian/Alaska Native	3 (0.1)	3 (0.1)	4 (0.2)	90 (0.4)	
Asian or Pacific Islander	110 (2.8)	53 (1.0)	30 (1.3)	1,102 (4.5)	
**MARITAL STATUS**, ***n*** **(%)**^a^					
Single	570 (15.2)	693 (13.7)	281 (12.4)	2,102 (17.0)	<0.001^*^
Married	3,183 (84.8)	4,360 (86.3)	1,979 (87.6)	10,295 (83.0)	
**INSURANCE RECODE**, ***n*** **(%)**^b^					
Uninsured	132 (4.6)	193 (5.1)	42 (2.6)	300 (3.3)	<0.001^*^
Any Medicaid	169 (5.9)	385 (10.2)	118 (7.2)	1,247 (13.6)	
Insured	2,549 (89.4)	3,188 (84.7)	1,480 (90.2)	7,645 (83.2)	
**Laterality**, ***n*** **(%)**					
Not a paired site	642 (16.1)	811 (15.6)	471 (20.3)	2,398 (18.8)	<0.001^*^
One side involvement	3,232 (81.3)	4,231 (81.2)	1,807 (77.9)	10,071 (79.0)	
Bilateral involvement	52 (1.3)	84 (1.6)	32 (1.4)	181 (1.4)	
Paired site	51 (1.3)	66 (1.7)	10 (0.4)	103 (0.8)	
**HISTOLOGICAL CLASSIFICATION**, ***n*** **(%)**					
Glioblastoma	3,858 (97.0)	5,056 (97.0)	2,236 (96.4)	1,320 (96.6)	0.057
Giant cell glioblastoma	32 (0.8)	34 (0.7)	21 (0.9)	144 (1.1)	
Gliosarcoma	87 (2.2)	122 (2.3)	63 (2.7)	289 (2.3)	
**EXTENT OF DISEASE**, ***n*** **(%)**^c^					
Localized	3,111 (82.7)	4,096 (81.2)	1,763 (78.5)	9,647 (78.2)	<0.001^*^
Regional	597 (15.9)	877 (17.4)	462 (20.6)	2,489 (20.2)	
Distant	53 (1.4)	72 (1.4)	20 (0.9)	208 (1.7)	
**TUMOR SIZE**, ***n*** **(%)**^d^					
<25 mm	383 (13.1)	448 (10.4)	208 (10.8)	1,050 (9.9)	<0.001^*^
25-49 mm	1,420 (48.4)	2,057 (47.9)	910 (47.2)	4,944 (46.7)	
50-99 mm	1,118 (38.1)	1,777 (41.4)	805 (41.7)	4,552 (43.0)	
≥100 mm	12 (0.4)	13 (0.3)	6 (0.3)	31 (0.3)	
**EXTENSION OF TUMOR**, ***n*** **(%)**^e^					
No	264 (7.0)	331 (6.6)	162 (7.2)	554 (4.5)	<0.001^*^
Yes	3,493 (93.0)	4,712 (93.4)	2,082 (92.8)	11,786 (95.5)	
**METASTASIS AT DIAGNOSIS**, ***n*** **(%)**^f^					
No	3,416 (98.7)	4,731 (98.7)	2,097 (99.1)	11,713 (95.8)	0.082
Yes	45 (1.3)	64 (1.3)	18 (0.9)	183 (1.5)	
**TREATMENTS**, ***n*** **(%)**^g^					
No surgery and radiation	391 (10.0)	612 (12.2)	237 (10.4)	1,720 (13.8)	<0.001^*^
Only surgery	548 (14.0)	790 (15.7)	302 (13.3)	2,053 (16.5)	
Only radiation	427 (10.9)	674 (13.4)	315 (13.8)	1,554 (12.5)	
Surgery+Radiation	2,545 (65.1)	2,950 (58.7)	1,442 (62.5)	7,123 (57.2)	

a *Unknown Marital status at diagnosis, n = 799*.

b *Data not applicable, n = 6,814*.

c *Unknown/Unstaged, n = 867*.

d *Unknown/Unstaged /Not applicable, n = 4,528*.

e *Unknown; extension not stated/ Primary tumor cannot be assessed/Not documented in patient record, n = 878*.

f *Unknown; distant metastasis not stated/Distant metastasis cannot be assessed/Not documented in patient record, n = 1,995*.

g *Unknown if surgery or radiation performed, n = 599*.

* *indicates a significant difference among the groups, P < 0.05*.

A choice of treatment plan: a surgery, radiotherapy or a combination of both varies between the evaluated regions. A total of 14,040 (57.9%) patients with glioblastoma underwent surgery and radiotherapy treatment, and only 2,960 (12.2%) patients did not have surgery and radiotherapy, while 3,693 (15.2%) patients from all regions evaluated underwent surgery exclusively. The highest rate of surgery followed by radiotherapy (65.1%) and lowest rate of radiotherapy alone (10.9%) was observed among patients from the Northeast region.

### Overall survival

**Figure 2:**
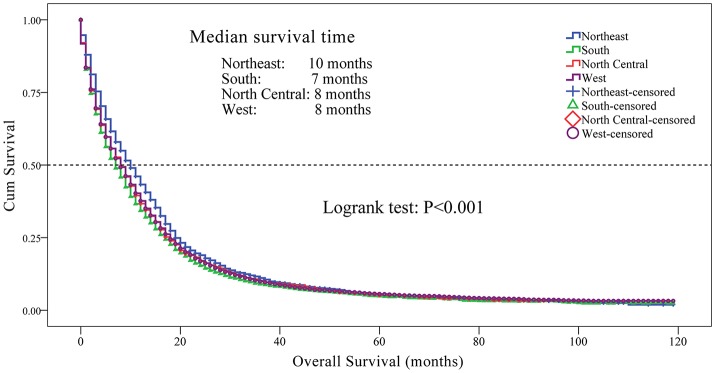
Kaplan-Meier curves for overall survival in patients with glioblastoma between 2004 and 2013 according to registry site. Overall survival of patients with glioblastoma from the Northeast region was longer compared to the North Central, West and the South regions (medium survival 10, 8, 8, and 7 months respectively; *P* < 0.001). The 1-, 3-, and 5-year survival rates for patients from different regions were as follows: Northeast—43.3, 11.1, 5.3%; South—34.3, 9.0, 4.9%; North Central—36.7, 10.3, 5.1%; and West—37.6, 9.9, 5.6%, respectively. Kaplan-Meier analysis and with log-rank test were performed using the statistical software package SPSS version 22.

We observed a significant difference in the OS rate in patients from different registry sites (Figure 2, *P* < 0.001). OS was longer in patients from the Northeast, followed by the North Central, West and South regions. We did not observe a significant difference in OS between patients from the North Central and West regions (*P* = 0.817). The median survival time was 10 months for patients from the Northeast, 8 months for patients from the North Central and West, and 7 months for patients from the South regions. The 1-, 3-, and 5-year survival rates for patients from different regions were as follows: Northeast-43.3, 11.1, and 5.3%; South - 34.3, 9.0, and 4.9%; North Central-36.7, 10.3, and 5.1%; and West-37.6, 9.9, and 5.6%, respectively.

### The risk of mortality among different prognostic elements

Age, gender, race, registry site, marital status, insurance recode, laterality, histological classification, the extent of disease, tumor size, an extension of tumor, metastasis, and treatments were identified as significant prognostic factors for overall survival mortality among individuals with glioblastoma in univariate analysis (*P* < 0.05, Table 2).

**Table 2:** Cox regression analyses of overall survival in patients with glioblastoma.

	**Crude HR (95% CI)**	***P*-value**	**Adjusted HR (95% CI)**	***P*-value**
Diagnostic age	1.036 (1.035, 1.037)	<0.001^*^	1.030 (1.028, 1.032)	<0.001^*^
**GENDER**				
Female vs. Male	1.059 (1.021, 1.080)	0.001^*^		
**RACE**				
Black vs. White	0.901 (0.849, 0.956)	0.001^*^	0.919 (0.843, 1.003)	0.057
American Indian/Alaska Native vs. White	0.883 (0.703, 1.110)	0.286	1.130 (0.841, 1.518)	0.417
Asian or Pacific Islander vs. White	0.797 (0.744, 0.855)	<0.001^*^	0.769 (0.699, 0.847)	<0.001^*^
**REGISTRY SITE**				
South vs. Northeast	1.175 (1.123, 1.230)	<0.001^*^	1.245 (1.163, 1.332)	<0.001^*^
North Central vs. Northeast	1.108 (1.047, 1.172)	<0.001^*^	1.167 (1.075, 1.267)	<0.001^*^
West vs. Northeast	1.102 (1.059, 1.146)	<0.001^*^	1.102 (1.038, 1.170)	<0.001^*^
**MARITAL STATUS**				
Married vs. Single	1.179 (1.133, 1.227)	<0.001^*^	0.905 (0.853, 0.959)	0.001^*^
**INSURANCE RECODE**				
Any Medicaid vs. Uninsured	1.174 (1.058, 1.301)	0.002^*^	1.061 (0.940, 1.198)	0.335
Insured vs. Uninsured	1.133 (1.033, 1.242)	0.008^*^	0.926 (0.831, 1.033)	0.170
**LATERALITY**				
One side involvement vs. Not a paired site	0.736 (0.711, 0.763)	<0.001^*^	0.858 (0.811, 0.907)	<0.001^*^
Bilateral involvement vs. Not a paired site	1.202 (1.071, 1.350)	0.002^*^	0.886 (0.748, 1.050)	0.164
Paired site vs. Not a paired site	0.846 (0.734, 0.974)	0.020^*^	1.027 (0.794, 1.327)	0.840
**HISTOLOGICAL CLASSIFICATION**				
Giant cell glioblastoma vs. Glioblastoma	0.685 (0.589, 0.795)	<0.001^*^		
Gliosarcomavs. Glioblastoma	0.907 (0.827, 0.995)	0.039^*^		
**EXTENT OF DISEASE**				
Regional vs. Localized	1.456 (1.405, 1.508)	<0.001^*^	1.383 (1.313, 1.456)	<0.001^*^
Distant vs. Localized	1.591 (1.419, 1.783)	<0.001^*^	1.500 (1.275, 1.766)	<0.001^*^
**TUMOR SIZE**				
25–49 vs. < 25 mm	1.049 (0.995, 1.105)	0.076	1.076 (1.007, 1.149)	0.030^*^
50–99 vs. < 25 mm	1.178 (1.114, 1.239)	<0.001^*^	1.206 (1.127, 1.291)	<0.001^*^
≥100 vs. < 25 mm	1.259 (0.953, 1.663)	0.105	1.291 (0.875, 1.906)	0.198
**EXTENSION OF TUMOR**				
Yes vs. No	0.903 (0.850, 0.959)	0.001^*^		
**METASTASIS AT DIAGNOSIS**				
Yes vs. No	1.500 (1.328, 1.693)	<0.001^*^		
**TREATMENT**				
Only surgery vs. no surgery and radiation	0.542 (0.515, 0.570)	<0.001^*^	0.630 (0.583, 0.681)	<0.001^*^
Radiation vs. no surgery and radiation	0.381 (0.361, 0.402)	<0.001^*^	0.403 (0.371, 0.437)	<0.001^*^
Surgery+Radiation vs. no surgery and radiation	0.200 (0.192, 0.209)	<0.001^*^	0.231 (0.215, 0.248)	<0.001^*^

* *indicates significant factors, P < 0.05*.

Multivariate Cox regression analysis demonstrated that aging (HR, 1.030; 95% CI, 1.028 to 1.032; *P* < 0.001), South registry site (HR, 1.245; 95% CI, 1.163 to 1.332; *P* < 0.001), North Central registry site (HR, 1.167; 95% CI, 1.075 to 1.267; *P* < 0.001), West registry site (HR, 1.102; 95% CI, 1.038 to 1.170; *P* < 0.001), extent of disease (HR, 1.383; 95% CI, 1.313 to 1.456; *P* < 0.001), distant extent of disease (HR, 1.500; 95% CI, 1.275 to 1.766; *P* < 0.001), tumor size between 25 and 49 mm (HR, 1.076; 95% CI, 1.007 to 1.149; *P* = 0.030), and tumor size between 50 and 99 mm (HR, 1.206; 95% CI, 1.127 to 1.291; *P* < 0.001) were associated with worse survival outcomes. However, Asian or Pacific Islander race (HR, 0.769; 95% CI, 0.699 to 0.847; *P* < 0.001), married status (HR, 0.905; 95% CI, 0.853 to 0.959; *P* = 0.001), and unilateral tumor location (HR, 0.858; 95% CI, 0.811 to 0.907; *P* < 0.001) were associated with the improved OS (Table 2). In addition, patients who underwent both surgery and radiation treatment had a lower risk of mortality compared to those who had either surgery or radiation therapy alone (HR: 0.231, 95% CI: 0.215 to 0.248, *P* < 0.001).

## Discussion

The aim of this study was to explore the regional incidence of glioblastoma in the USA during 2004–2013 and determine the prognostic factors in glioblastoma patients. We found that the glioblastoma incidence differed among examined US regions, with the highest incidence rate among the patients from the South registry site. In addition, South registry site had the strongest association with increased mortality compared to other regions.

Furthermore, we observed statistically significant differences in age, race, marital status, insurance recode, laterality, extent of disease, tumor size, extension of tumor, and treatments among patients from different regions. In agreement with previous studies, we demonstrated that age, race, extent of disease, tumor size, and treatment plan were prognostic factors for survival outcome in a multivariate analysis. To our knowledge, this is one of the largest and the most up to date studies examining glioblastoma incidence from the geographic point and factors influencing its outcome.

Glioblastoma is the most common type of glioma, which accounts for up to 77–81% of all primary malignant tumors of CNS (Schwartzbaum et al., 2006; Ostrom et al., 2014). Many reports are based on statistics, which reflect the incidence of primary malignancies of the nervous system in general, thus providing a limited representation of glioblastoma distribution. However, such studies aid in the identification of potential factors associated with the disease and population at risk. Such is the study by Ostrom et al, who reported the highest incidence of primary malignant tumors of the nervous system in the northeast while the south-central regions of the US had the lowest incidence (Ostrom et al., 2014). Seemingly contradictive with our findings, these data point to the fact that careful consideration should be given when trying to infer information about glioblastoma distribution using less specific statistics. Geographic variations in glioblastoma incidence were published in previous reports. Devesa et al reported the geographic variation in the incidence of brain cancer and various cancers of the nervous system in the United States (Devesa et al., 1999). Authors found higher incidence rates of the named diseases in the southeast, northwest, and midwest, and lower rates in the Rocky Mountains, northeast, and southwest. Efird et al. reported that in the United States, the incidence rate (IR) per 100,000 person-years (100KP-Y) for malignant adult brain tumors ranges from 5.4 for the state of Hawaii to 12 for Wisconsin (Efird, 2011). The highest age-adjusted incidence and death rates (DR) per 100KP-Y were observed in Kentucky (7.9), Iowa (7.6), and Oregon (IR = 7.5). According to CBTRUS 2005–2006 Statistical Report: Primary Brain Tumors in the United States, 1998–2002, the average annual age-adjusted incidence rate of primary malignant brain and CNS tumors in adults ranged from 7.3 per 100,000 person-years in Virginia to 10.5 per 100,000 person-years in Maine and Idaho (ICBTRotUS, 2012). Despite the fact that the above statistics account for numerous types of neurological malignancies and is not immediately reflective of the incidence rate and geographical distribution of glioblastomas, the data are important in demonstrating the regional differences in the incidence rates of the brain malignancies in adults.

One of the factors contributing to the regional differences in tumor incidence is an overall access to health care (Wrensch et al., 2002). A number of studies showed that rural areas had fewer providers and hospitals than urban areas (Reschovsky and Staiti, 2005), leading to limited access to healthcare, and higher healthcare cost (Hartley, 2004). Variations in diagnostic practices and comprehensiveness of reporting can also contribute to what appears as geographic differences in the incidence rate (Wrensch et al., 2002).

The role of environmental factors and the patient's lifestyle in the geographic variations of the incidence rate also cannot be excluded. Multiple environmental factors, including diet, occupational and personal exposures and lifestyle have been evaluated in an attempt to find a statistically significant association with disease and provided inconclusive outcomes. However, an inverse association has been demonstrated between glioma incidence and prior history of allergies and infectious diseases (Miranda-Filho et al., 2017).

In addition, ethnic/race variations are likely to contribute to observed differences (Barnholtz-Sloan et al., 2003, 2007). For example, it was shown that the Black, Asian and Hispanic patients had a significantly lower risk of mortality and improved survival compared to non-Hispanic Caucasian patients (Gabriel et al., 2014; Pan et al., 2015). Several genetic susceptibility loci for glioma were identified in genome-wide association studies (Shete et al., 2009; Wrensch et al., 2009). It is possible that due to genetic variability across the race/ethnic groups (Genomes Project et al., 2010), the frequency of susceptibility alleles also varies and may contribute to differences in the glioma incidence. Furthermore, several studies have identified race-specific genetic aberrations in glioma (Mochizuki et al., 1999; Chen et al., 2001; Das et al., 2002). Detection of additional glioblastoma genetic predisposition factors will aid in understanding the mechanisms of this disease.

We observed statistically significant differences in age, race, marital status, insurance recode, laterality, the extent of disease, tumor size, an extension of tumor, and treatments among patients from different regions. In agreement with previous studies, we showed that age, race, the extent of disease, tumor size, and treatment type were prognostic factors for survival outcome in a multivariate analysis (Ostrom et al., 2014).

This study provides the most up to date large-scale examination of glioblastoma incidence with respect to the geographic location and factors influencing the disease outcome.

The strengths and limitations of this study arise from the usage of SEER database as a data source. SEER database is comprehensive and allows essentially complete assessment of cancer cases from the source population with limited selection bias. Data derived from SEER include information on various tumor characteristics, follow-ups for vital status and cause of death. In addition, cancer registries participating in the SEER program are required to meet strict quality control requirements with respect to case ascertainment and data quality (http://seer.cancer.gov). Limitations include lack of randomization, information on comorbidities, and lifestyle factors. Besides, information and details of chemotherapy and immunotherapy are not reported in SEER database.

The vast majority of glioblastoma cases are of unknown cause. Variations in glioblastoma incidence between different races and geographic locations point out to the genetic and environmental risk factors. However, they can also be explained by differences in health care quality and access, study bias, and other unknown factors. It is also likely that multiple factors interact to influences the development of glioblastoma in a given individual, and effects of individual factors might not be apparent when examined in isolation. Therefore, future studies with improved methods to assess potential contributing factors and more precise statistical methods for detecting interaction effects, are warranted for a better understanding of glioblastoma development and identification of at-risk populations.

Despite the complexity of the problem, we believe that identification of geographic areas associated with increased glioblastoma incidences and poorer outcomes can promote awareness and may result in improved protocols for glioblastoma detection and patient care in high-risk regions.

## Conclusions

Our study highlights that glioblastoma incidences are geographic region and race/ethnicity- dependent. Specifically, we showed that in the US the highest incidence rate was among patients from the South region. In addition, South registry sites region had the strongest association with increased mortality. Multivariate Cox regression analysis demonstrated that overall survival was better in patients of Asian or Pacific Islander race. In addition, we observed statistically significant differences in age, marital status, insurance status, tumor laterality, the extent of disease, tumor size, an extension of tumor, and treatment protocol types among patients from different regions. Results of our study improve understanding of regional differences in glioblastoma incidence and pave the road for identification of the regional risk factors which should lead to improved protocols for glioblastoma detection, prevention, and management.

## Author contributions

HaX: literature research, data acquisition, data analysis, and statistical analysis. JC: literature research, data acquisition, and manuscript review; HoX: literature research and manuscript review; ZQ: guarantor of integrity of the entire study, study design, manuscript editing, and manuscript review.

### Conflict of interest statement

The authors declare that the research was conducted in the absence of any commercial or financial relationships that could be construed as a potential conflict of interest.
